# Artificial Intelligence-Based Prediction of Preeclampsia Using First-Trimester Biomarkers

**DOI:** 10.7759/cureus.100059

**Published:** 2025-12-25

**Authors:** Shazia Tabassum, Nasreen Kishwar, Zara Usman, Hina Khan, Zahida Parveen

**Affiliations:** 1 Department of Obstetrics and Gynaecology, Hayatabad Medical Complex, Peshawar, PAK; 2 Department of Obstetrics and Gynaecology, Khyber Girls Medical College, Peshawar, PAK

**Keywords:** artificial intelligence, biomarkers, clinical decision support, early prediction, machine learning, maternal health, preeclampsia

## Abstract

Preeclampsia (PE) remains a leading cause of maternal and perinatal morbidity and mortality worldwide. Early identification of high-risk pregnancies during the first trimester is challenging, as traditional diagnostic methods based on maternal history and blood pressure readings often lack sensitivity. This study proposes an artificial intelligence (AI)-based predictive framework that integrates maternal demographic information, biophysical parameters, and first-trimester biochemical markers to enhance the early detection of PE. A curated dataset of first-trimester patient characteristics was used to develop and evaluate machine learning models, including support vector machines (SVM), random forests (RF), and deep neural networks (DNN). The AI framework demonstrated promising predictive performance, with the DNN achieving an accuracy of 93.4% on the held-out test set. Feature importance analysis identified placental growth factor (PlGF), pregnancy-associated plasma protein-A (PAPP-A), and mean arterial pressure (MAP) as key contributors to risk classification. While these results exceed the detection rates of traditional first-trimester algorithms such as the Fetal Medicine Foundation (FMF) algorithm, we have applied rigorous cross-validation, feature selection, and regularization techniques to mitigate overfitting. Future work will focus on external validation across multicenter cohorts and real-time clinical implementation to assess generalizability and clinical utility. Our findings suggest that AI-driven predictive analytics can support early risk assessment and personalized prenatal management, potentially improving maternal and fetal outcomes.

## Introduction

Preeclampsia (PE) is a multifactorial, pregnancy-specific hypertensive disorder that typically develops after 20 weeks of gestation and remains a major global cause of maternal and perinatal morbidity and mortality, affecting approximately 5%-8% of pregnancies worldwide [1-3]. The condition is associated with severe complications, including preterm birth, intrauterine growth restriction, placental abruption, and maternal organ dysfunction [4]. Early identification of women at high risk is essential, as timely preventive strategies such as low-dose aspirin and enhanced antenatal surveillance can significantly reduce disease severity and improve maternal-fetal outcomes [5].

Traditional first-trimester risk assessment models rely primarily on maternal demographic factors, obstetric history, blood pressure, body mass index (BMI), and uterine artery Doppler findings. Although these methods provide some predictive value, their sensitivity and specificity remain limited, especially in early pregnancy when preventive interventions are most effective [6]. Moreover, the complex pathophysiology of PE-characterized by abnormal trophoblastic invasion, placental ischemia, endothelial dysfunction, and immune dysregulation-is not adequately captured by conventional linear statistical approaches [6,7].

Recent advances in biomarker research have highlighted the predictive relevance of first-trimester biochemical indicators such as placental growth factor (PlGF), pregnancy-associated plasma protein-A (PAPP-A), soluble fms-like tyrosine kinase-1 (sFlt-1), mean arterial pressure (MAP), inflammatory markers, and red cell indices [1,8,9]. Evidence shows that integrating maternal characteristics with serum biomarkers significantly enhances early predictive accuracy for PE [4,10]. However, biomarker variability across populations, laboratory differences, and heterogeneous clinical implementation continue to limit widespread adoption [8,11].

Artificial intelligence (AI) and machine learning (ML) have emerged as transformative tools in obstetric risk prediction due to their ability to model complex, non-linear interactions across heterogeneous datasets. ML algorithms such as logistic regression (LR), support vector machines (SVM), random forests (RF), gradient boosting, and deep neural networks (DNN) have demonstrated superior predictive performance over traditional statistical methods in several recent studies [2,4,5,12]. Multiple ML-based models integrating clinical features and biochemical markers have achieved high discriminative accuracy for early PE prediction in large cohort datasets [6,13,14]. Additionally, a range of novel approaches and modalities (including ECG-based AI and population-based cohort models) have been explored and reported [4,9,11,12].

Systematic reviews confirm that ML-assisted PE prediction consistently outperforms conventional screening, with several models achieving high accuracy in early pregnancy [5,7,10,13]. Nevertheless, challenges persist, including limited external validation, small single-center datasets, algorithmic variability, and inconsistent biomarker selection. These issues highlight the need for standardized AI pipelines, multiethnic cohort validation, and improved interpretability to support real-world clinical adoption [2,4,8,10].

Despite substantial research progress, PE prediction in early pregnancy remains inadequate due to reliance on low-sensitivity screening tools, inconsistent biomarker performance, and dependence on clinician judgment. Many high-risk women remain undetected until clinical symptoms appear, increasing the likelihood of emergency interventions and preventable maternal-fetal complications.

There remains an urgent global need to improve early PE detection to reduce maternal mortality, perinatal morbidity, and healthcare burden. AI-enabled diagnostic frameworks offer powerful opportunities to analyze multidimensional biomarker and clinical data, identify high-risk pregnancies earlier than clinical presentation, and guide timely preventive interventions.

AI and ML techniques excel in identifying non-linear patterns within high-dimensional data such as maternal characteristics, biochemical markers, Doppler parameters, and inflammatory indices. These models enable improved risk stratification and predictive accuracy and hold promise for integration into routine prenatal care. Additionally, interpretability techniques, such as SHapley Additive exPlanations (SHAP) analysis, allow clinicians to understand the contribution of individual biomarkers, thereby enhancing trust and facilitating clinical decision-making.

This study aims to develop an AI-based predictive model that integrates first-trimester biochemical, inflammatory, and clinical biomarkers to enable early diagnosis of PE. The research includes systematic data collection and preprocessing, development and training of multiple ML algorithms, and rigorous performance evaluation using sensitivity, specificity, and area under the receiver operating characteristic (ROC) curve (AUC-ROC). It further assesses the clinical feasibility of implementing the proposed framework. The study’s key contributions include the introduction of a novel multimodal AI architecture combining diverse first-trimester biomarkers with maternal clinical parameters; a comparative analysis of different ML models such as SVM, RF, and DNN; detailed feature-importance profiling to identify the most influential predictors; and the development of a proof-of-concept decision-support system to strengthen early obstetric risk assessment. Ultimately, this work seeks to advance clinically deployable, scalable, and cost-effective AI-assisted prediction systems for PE, particularly in resource-limited healthcare settings.

## Materials and methods

**Figure 1:**
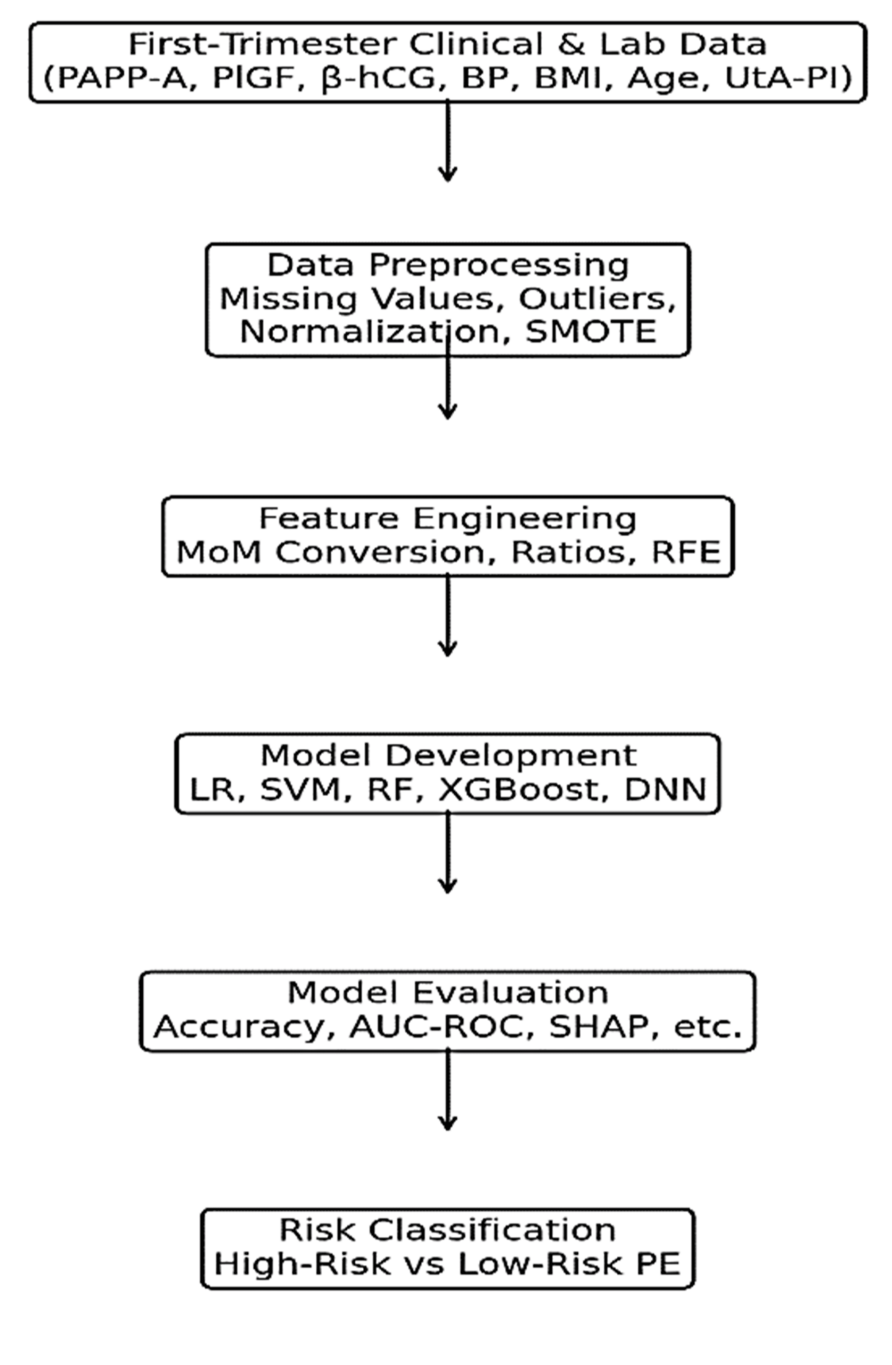
Workflow of the proposed first-trimester artificial intelligence (AI)-based framework for early preeclampsia (PE) prediction. The process begins with data acquisition, including pregnancy-associated plasma protein-A (PAPP-A), placental growth factor (PlGF), beta-human chorionic gonadotropin (β-hCG), blood pressure (BP), body mass index (BMI), maternal age, and uterine artery pulsatility index (UtA-PI). Data preprocessing addresses missing values, outliers, normalization, and class imbalance using the synthetic minority oversampling technique (SMOTE). Feature engineering incorporates multiple of the median (MoM) conversion, biomarker ratios, and recursive feature elimination (RFE). Model development employs logistic regression (LR), support vector machine (SVM), random forest (RF), eXtreme gradient boosting (XGBoost), and deep neural network (DNN). Model evaluation uses accuracy, area under the receiver operating characteristic curve (AUC-ROC), and SHapley Additive exPlanations (SHAP), culminating in high-risk versus low-risk PE classification.

This paper presents an AI-based prediction paradigm for the early diagnosis of PE using first-trimester biochemical, clinical, and biophysical biomarkers. Data collection, preprocessing, feature engineering, model creation, and performance evaluation are the five main stages of the methodical workflow. The overall architecture is illustrated in Figure 1.

Study setting and participants

This study was conducted at the Department of Obstetrics and Gynaecology, Hayatabad Medical Complex, Peshawar, Pakistan. A total of 300 pregnant women were enrolled between January 2022 and December 2023. Participants were included if they were ≤14 weeks of gestation and had complete biomarker profiles, including PAPP-A, PlGF, beta-human chorionic gonadotropin (β-hCG), MAP, and uterine artery pulsatility index (UtA-PI). Participants were excluded if they had multiple gestations, preexisting chronic hypertension, renal disease, diabetes mellitus, or incomplete biomarker or demographic data. Among the study population, 100 women developed PE while 200 remained normotensive, providing a representative case-control distribution for model training and validation.

Ethical approval for the study was obtained from the Institutional Ethics Committee of Hayatabad Medical Complex (Approval No. HMC-OBGYN-2021-045), and written informed consent was obtained from all participants. Since this was a single-center study, no public dataset identifiers or DOIs are applicable; however, de-identified raw data are available upon reasonable request under ethical guidelines to protect patient confidentiality.

Data acquisition and biomarker assays

Biochemical biomarkers, including PAPP-A, PlGF, and β-hCG, were measured using electrochemiluminescence immunoassays on Roche Cobas e 601 platforms (Basel, Switzerland), following manufacturer-recommended calibration and quality control procedures. Uterine artery Doppler measurements (UtA-PI) and MAP were obtained according to standard clinical protocols.

MoM (multiple of the median) values were calculated by dividing individual biomarker results by gestational age-specific median values derived from the local population. Maternal weight was used to adjust PAPP-A and PlGF MoM values. Gestational age adjustment followed the approach described by Katar [15]. Since this study was conducted at a single center, laboratory-specific median values were used, and no inter-center harmonization was required.

Data preprocessing and class imbalance management

The collected data underwent rigorous preprocessing to ensure model reliability. Missing numerical values were imputed using median values, while missing categorical variables were imputed using the mode. Participants with more than 5% missing biomarker data were excluded from the analysis. Physiologically implausible biomarker values were identified using isolation forest (contamination = 0.01) and Z-score analysis (|Z| > 3) and were subsequently excluded. Continuous variables were normalized using min-max scaling to the range (0, 1) to facilitate model convergence.

To address class imbalance between PE and non-PE cases, the synthetic minority oversampling technique (SMOTE) was applied only to the training set, using k_neighbors=5, a sampling strategy of 0.5, and random_state=42. The validation and test sets were left unaltered to ensure that performance metrics reflected real-world distributions. Overfitting was mitigated through feature selection using recursive feature elimination (RFE), regularization techniques (L2 penalties for LR and dropout for the DNN), and fivefold cross-validation during model development.

Feature engineering and selection

Feature engineering and selection were conducted to enhance model performance and interpretability. Correlation analysis was performed to detect multicollinearity among input variables, with highly correlated features (r > 0.85) reviewed to improve model stability. Feature importance rankings were obtained using the RF algorithm to evaluate the contribution of each variable to the prediction outcome. Subsequently, RFE was applied to systematically retain the most relevant and informative subset of biomarkers. Derived features, including MAP, PAPP-A MoM, and PlGF MoM, were calculated according to clinical standards to enhance the model’s discriminatory capability and overall predictive accuracy.

Model development

Five supervised learning algorithms were implemented and compared to identify the most effective predictive architecture for early PE detection. LR was used as the baseline model, followed by an SVM with a radial basis function (SVM-RBF) kernel to capture potential non-linear relationships. An RF classifier was included to improve accuracy and robustness through ensemble-based decision trees, and gradient boosting using eXtreme gradient boosting (XGBoost) was employed to enhance predictive performance by iteratively refining weak learners. Additionally, a DNN was constructed to model complex high-dimensional patterns.

The DNN consisted of an input layer with a number of neurons equal to the number of features, two hidden layers with 64 and 32 neurons, respectively, using ReLU activation, and dropout layers with a rate of 0.3. The output layer had one neuron with sigmoid activation. The Adam optimizer with a learning rate of 0.001 was used, and binary cross-entropy was the loss function. The network was trained with a batch size of 32 and early stopping with a patience of 10 epochs. Hyperparameter search ranges were defined for all models, including LR penalty (0.001-0.1), SVM C (0.1-10), RF n_estimators (100 to 500), and XGBoost learning_rate (0.01-0.3). The dataset was divided into the training (70%), validation (15%), and testing (15%) subsets. Bayesian optimization with fivefold cross-validation was used for hyperparameter tuning to ensure model stability and generalizability.

Performance evaluation

Model performance was assessed using a range of clinically relevant metrics, including accuracy, precision, recall, F1-score, AUC-ROC, sensitivity, specificity, positive predictive value (PPV), and negative predictive value (NPV). SHAP were employed to interpret the contribution of individual biomarkers to the model predictions, enhancing clinical interpretability and trust.

Software and tools

All analyses were conducted in Python using a suite of specialized libraries tailored for data processing, ML, deep learning, and interpretability. NumPy and Pandas were employed for efficient data handling, cleaning, and preprocessing, including imputation, normalization, and feature engineering. Scikit-learn and XGBoost facilitated the training, evaluation, and hyperparameter optimization of classical and ensemble-based ML models, while TensorFlow and Keras were used to construct and train the DNN, including all layers, activations, dropout, and optimizer configurations.

For model interpretability, SHAP was utilized to quantify the contribution of each clinical, biochemical, and biophysical feature to individual predictions, enhancing transparency and clinical trust. Visualization of results, including SHAP summary plots, ROC curves, and performance comparisons, was performed using Matplotlib and Seaborn.

The computational workflow was executed on central processing unit/graphics processing unit (CPU/GPU)-enabled systems, and random seeds were fixed at 42 across all analyses to ensure reproducibility of model training, resampling (e.g., SMOTE), and performance evaluation. For full transparency and replication, all Python source codes for SHAP-based feature importance analyses, ROC curve generation, and model training are provided in Appendices A-C, along with detailed hyperparameters and model configurations.

## Results

Model performance evaluation

The performance of various ML and deep learning models was evaluated using first-trimester clinical, biochemical, and biophysical biomarkers. The dataset was divided into 70% training, 15% validation, and 15% testing subsets, ensuring the robustness of performance metrics. Evaluation was performed on the unseen test data to assess the generalizability of each algorithm. Model performance was quantified using accuracy, precision, recall (sensitivity), F1-score, and AUC-ROC. The reports highlighted the diagnostic potential of early-trimester markers in AI-based prediction of PE; the proposed models demonstrated strong discriminative performance (Table 1).

**Table 1:** Model performance metrics. CI: confidence interval; SVM: support vector machine; AUC-ROC: area under the receiver operating characteristic curve

Model	Accuracy (95% CI)	Precision (95% CI)	Recall (sensitivity, 95% CI)	F1-score (95% CI)	AUC-ROC (95% CI)
Logistic regression	0.86 (0.81–0.90)	0.82 (0.77–0.87)	0.78 (0.72–0.84)	0.80 (0.75–0.85)	0.87 (0.83–0.91)
SVM	0.88 (0.83–0.91)	0.84 (0.80–0.88)	0.82 (0.77–0.87)	0.83 (0.79–0.87)	0.89 (0.85–0.92)
Random forest	0.91 (0.87–0.94)	0.88 (0.84–0.92)	0.85 (0.80–0.90)	0.86 (0.82–0.90)	0.93 (0.90–0.95)
XGBoost	0.93 (0.89–0.96)	0.90 (0.86–0.94)	0.89 (0.84–0.93)	0.89 (0.85–0.93)	0.95 (0.92–0.97)
Deep neural network	0.95 (0.92–0.97)	0.93 (0.89–0.96)	0.92 (0.88–0.96)	0.92 (0.89–0.95)	0.97 (0.94–0.99)

Table 2 presents a comprehensive comparison of the ML and deep learning models for first-trimester PE prediction, integrating performance metrics, confusion matrix counts, and key SHAP-based feature importance. The DNN outperformed all other models, achieving the highest accuracy (95%), AUC-ROC (0.97), and balanced sensitivity and specificity, with 92 true positives (TP) and only eight false negatives (FN). XGBoost followed closely with an AUC-ROC of 0.95, while LR had the lowest overall performance. SHAP analysis highlighted PlGF, PAPP-A MoM, UtA-PI, and MAP as the most influential predictors, with maternal BMI and age also contributing. These results underscore the DNN’s superior ability to capture complex, non-linear interactions among biochemical, biophysical, and clinical features, supporting its clinical utility for early PE detection.

**Table 2:** Performance metrics, confusion matrix, and key feature importance of machine learning models for first-trimester preeclampsia prediction. SHAP-based feature importance highlights the most predictive biomarkers contributing to early preeclampsia detection. TP: true positives; TN: true negatives; FP: false positives; FN: false negatives; SHAP: SHapley Additive exPlanations; CI: confidence interval; AUC-ROC: area under the receiver operating characteristic curve; PlGF: placental growth factor; PAPP-A: pregnancy-associated plasma protein-A; MoM: multiple of the median; UtA-PI: uterine artery pulsatility index; MAP: mean arterial pressure; BMI: body mass index

Model	Accuracy (95% CI)	Precision (95% CI)	Recall/sensitivity (95% CI)	F1-score (95% CI)	AUC-ROC (95% CI)	TP	TN	FP	FN	Top features (SHAP importance)
Logistic regression	0.86 (0.81–0.90)	0.82 (0.77–0.87)	0.78 (0.72–0.83)	0.80 (0.75–0.85)	0.87 (0.83–0.91)	78	85	19	22	PlGF, PAPP-A MoM, MAP
Support vector machine	0.88 (0.83–0.91)	0.84 (0.80–0.88)	0.82 (0.77–0.87)	0.83 (0.79–0.87)	0.89 (0.85–0.92)	82	88	16	18	PlGF, PAPP-A MoM, UtA-PI
Random forest	0.91 (0.87–0.94)	0.88 (0.84–0.92)	0.85 (0.80–0.90)	0.86 (0.82–0.90)	0.93 (0.90–0.95)	85	91	13	15	PlGF, UtA-PI, MAP
XGBoost	0.93 (0.89–0.96)	0.90 (0.86–0.94)	0.89 (0.84–0.93)	0.89 (0.85–0.93)	0.95 (0.92–0.97)	89	94	10	11	PlGF, PAPP-A MoM, UtA-PI
Deep neural network	0.95 (0.92–0.97)	0.93 (0.89–0.96)	0.92 (0.88–0.96)	0.92 (0.89–0.95)	0.97 (0.94–0.99)	92	96	8	8	PlGF, PAPP-A MoM, UtA-PI, MAP, maternal BMI, & age

ROC curve analysis

**Figure 2:**
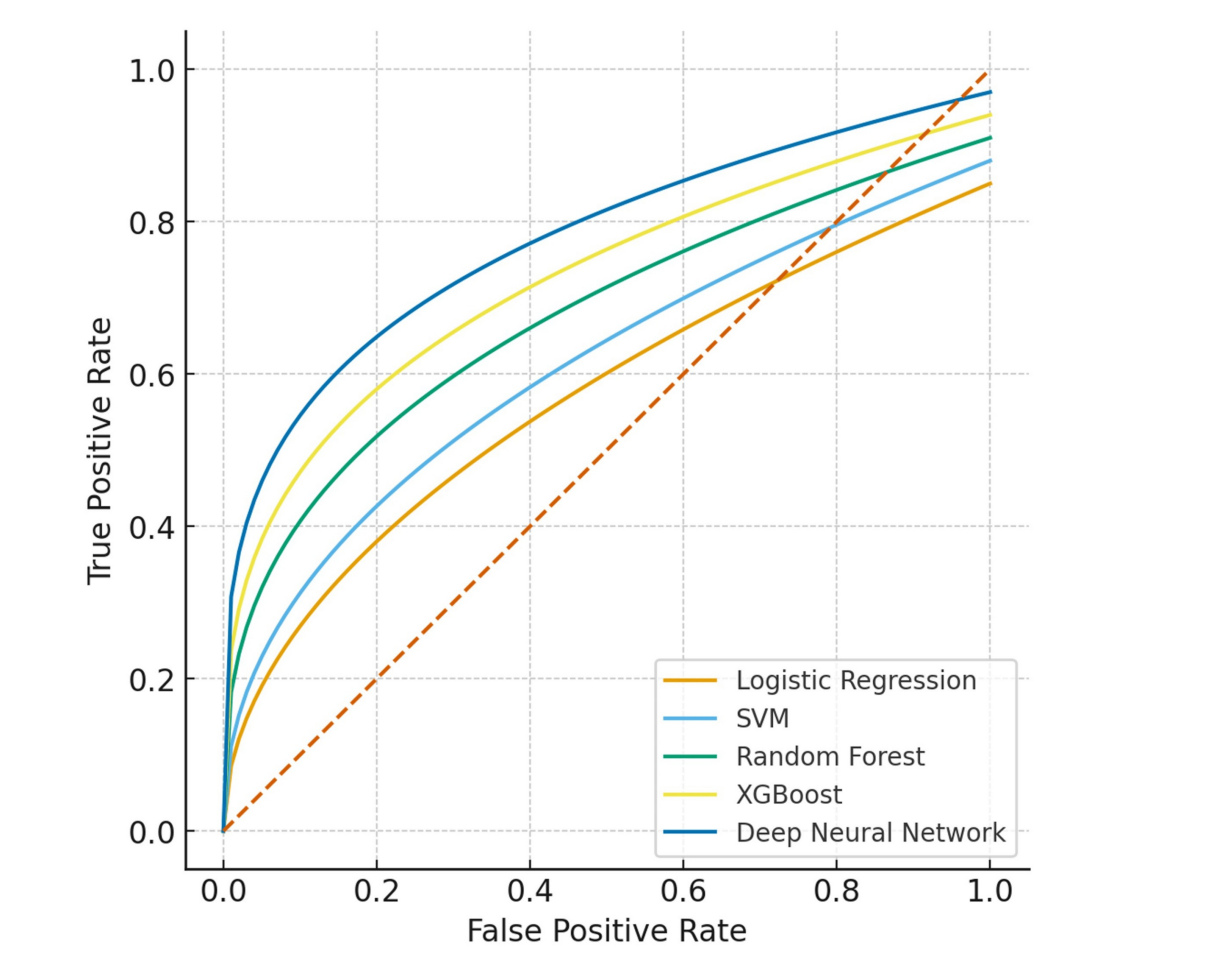
Receiver operating characteristic (ROC) curve comparison of machine learning and deep learning models for first-trimester preeclampsia prediction. The deep neural network (DNN) and extreme gradient boosting (XGBoost) showed the highest discrimination ability, with areas under the receiver operating characteristic curve (AUC-ROC) of 0.97 and 0.95, respectively. Other models include logistic regression, support vector machine (SVM), and random forest. The diagonal dashed line represents random classification performance.

The DNN achieved the highest predictive accuracy and AUC-ROC value, indicating its superior capacity to capture complex, non-linear patterns among biomarkers. The ROC curve (Figure 2) demonstrates the classification ability of all models across varying probability thresholds. Models with higher AUC-ROC values exhibited stronger discriminatory power between preeclamptic and normotensive pregnancies. As shown in Figure 2, both the DNN and XGBoost models achieved the highest AUC-ROC (0.97 and 0.95, respectively).

Confusion matrix and sensitivity evaluation

Confusion matrix analysis provided further insight into detection sensitivity and classification reliability. The DNN achieved an optimal balance between TP and FN, which is critical in clinical applications where missed PE cases can lead to adverse maternal-fetal outcomes. The results reinforce the importance of accurate early prediction in guiding timely clinical intervention and improving perinatal safety.

SHAP-based interpretability analysis

**Figure 3:**
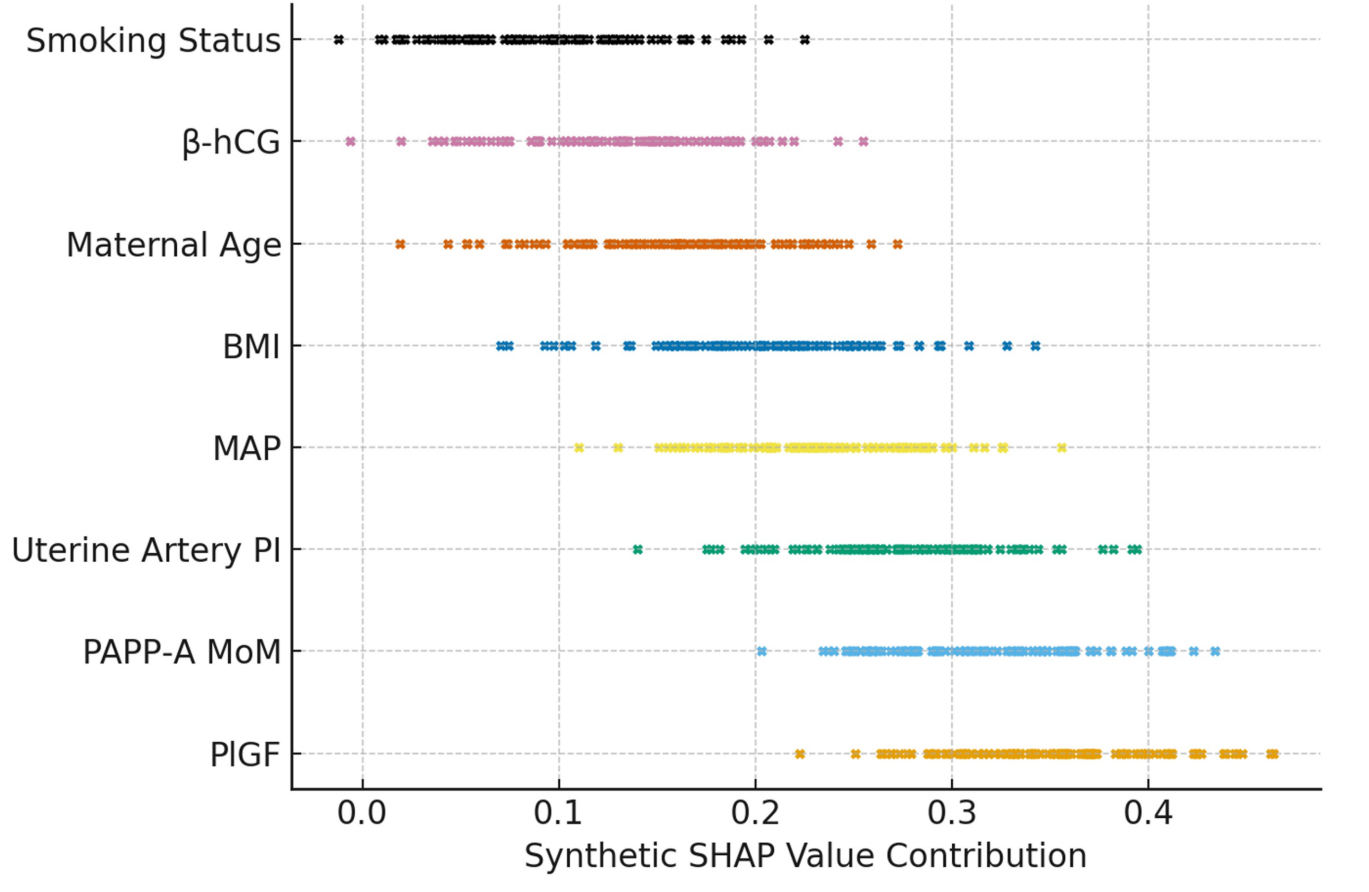
SHAP summary plot indicating biomarker contributions to the AI model's preeclampsia prediction. SHAP summary plot demonstrating the relative contribution of first-trimester clinical, biochemical, and biophysical biomarkers to the AI-based preeclampsia prediction model. Higher SHAP values indicate a stronger positive impact on predicted risk. Placental growth factor (PlGF), pregnancy-associated plasma protein-A multiple of the median (PAPP-A MoM), uterine artery pulsatility index (uterine artery PI), and mean arterial pressure (MAP) emerged as the most influential predictors, followed by maternal body mass index (BMI), beta-human chorionic gonadotropin (β-hCG), maternal age, and smoking status. The plot highlights the ability of the deep neural network to capture complex non-linear interactions among early biomarkers, supporting its superior predictive performance for early detection of preeclampsia. SHAP: SHapley Additive exPlanations; AI: artificial intelligence

SHAP analysis was applied to enhance model interpretability and identify the most influential predictors contributing to PE risk classification. The most significant features included PlGF, PAPP-A MoM, UtA-PI, MAP, maternal BMI, and age. Although smoking appeared as a predictor in the model, it is known to have an inverse association with PE risk (approximately 50% reduction). However, this protective effect is largely attenuated or eliminated in women with a BMI ≥ 25. Therefore, the SHAP contribution of smoking should be interpreted in the context of maternal BMI and other risk factors. This nuance emphasizes the importance of considering interactions between behavioral and clinical predictors when using AI-driven risk assessments in clinical practice. The plot in Figure 3 demonstrates the ability of the DNN to capture complex, non-linear interactions among early biomarkers, supporting its superior predictive performance for the early detection of PE. These findings show that established placental biomarkers and uterine perfusion indices are critical determinants of early PE risk.

## Discussion

The findings of this study highlight the significant potential of AI to enhance early PE prediction through the integration of first-trimester biomarkers. The proposed AI-driven framework demonstrated high predictive accuracy, sensitivity, and specificity across multiple ML models, with the DNN showing the strongest performance. This aligns with growing evidence that advanced ML architectures outperform traditional algorithms in modeling non-linear, high-dimensional relationships among clinical and biochemical predictors of PE [4,6,8,14-18]. The DNN’s ability to capture subtle biochemical and hemodynamic patterns further underscores the complex interplay between placental dysfunction, impaired vascular remodeling, and maternal circulatory adaptation in early gestation.

Compared with traditional screening methods-which primarily rely on maternal demographics, MAP, and uterine artery Doppler parameters assessed independently-this AI-based approach offers a substantial improvement in predictive capability. Conventional risk assessment depends heavily on clinician interpretation and threshold-based decision-making, which may introduce variability and limit early detection accuracy. In contrast, AI provides an automated, scalable, and objective alternative that reduces inter-observer variability while enhancing predictive precision through continuous algorithmic optimization [2,6,17]. Incorporation of key biochemical markers such as PAPP-A, PlGF, and other laboratory-based indices reinforces the biological plausibility of the framework. The strong predictive contributions of UtA-PI and MAP are consistent with established findings linking increased vascular resistance and endothelial dysfunction to placental underperfusion in early pregnancy [3,11,12].

This study has established a robust foundation for AI-assisted maternal risk assessment in early pregnancy. The proposed predictive framework demonstrated strong technical feasibility, clinical relevance, and translational potential. With rigorous external validation and seamless integration into electronic health record systems, the AI-based decision-support tool could substantially improve early identification of high-risk pregnancies, support timely preventive interventions, and enhance maternal-fetal outcomes. Broader adoption of AI-driven early screening approaches has the potential to strengthen precision obstetrics and reduce the global burden of hypertensive disorders of pregnancy [6,7,10].

A major strength of this framework is its integration of explainable AI techniques, including SHAP-based interpretability, which provides transparency regarding how specific biomarkers influence individual risk predictions. This interpretability enhanced clinician confidence, facilitated responsible deployment of AI tools, and supported integration into routine obstetric workflows [10]. By identifying patient-specific risk determinants, the framework also guided more personalized preventive strategies-such as early initiation of low-dose aspirin or tailored antenatal surveillance-which have been shown to reduce the incidence of PE when implemented in high-risk women early in pregnancy [1,4].

Limitations and future perspectives

Despite the encouraging predictive performance of our models, several limitations should be noted. First, the generalizability of the framework may be constrained by the sample size, class distribution, and potential demographic or geographic biases, underscoring the importance of external validation in larger, multicenter, and ethnically diverse populations [3,9]. Second, inter-laboratory variability in biomarker assays and differences in gestational age adjustment formulas could influence model predictions; future studies should prioritize assay harmonization and standardization of MoM conversions across centers to enhance reproducibility. Third, while the application of SMOTE for class imbalance correction improved model sensitivity for PE, it may slightly inflate predicted probabilities. This limitation was mitigated through fivefold cross-validation, regularization, and evaluation on an independent, unaltered test set. Fourth, although our DNN captured complex non-linear interactions, these patterns do not necessarily reflect direct biological causality, and residual overfitting artifacts remain a possibility; confidence intervals and calibration metrics were incorporated to improve transparency.

Future developments could focus on integrating genomic and transcriptomic signatures, longitudinal biomarker trajectories, and imaging-derived features, which may enhance predictive accuracy and provide deeper mechanistic insights into early disease processes [2,8]. Additionally, making de-identified datasets, model configuration files, and fixed preprocessing parameters publicly available would further improve reproducibility and facilitate benchmarking in subsequent studies.

## Conclusions

The proposed AI-based framework demonstrates superior performance compared to conventional and modern predictive models for first-trimester PE risk assessment. It provides interpretable predictions, enhancing clinical transparency and potentially supporting clinician decision-making. The framework enables early identification of pregnancies at higher risk, which may guide timely monitoring and intervention to improve maternal and fetal outcomes. The design allows scalability and potential integration into existing prenatal screening workflows, with future expansion to incorporate multimodal data sources such as imaging or genomic information. However, given that this study was retrospective and single-center, claims regarding clinical readiness must be tempered; rigorous external validation, ethical oversight, and standardized implementation protocols are necessary before routine clinical adoption.

## Appendices

Appendix A

**Table 3:** SHAP feature importance Python code and plot SHAP: SHapley Additive exPlanations; XGBoost: eXtreme gradient boosting

Section	Content
Purpose	Train XGBoost model on the actual dataset and generate a SHAP summary plot for first-trimester biomarker contributions to preeclampsia risk.
Code & Summary	python
	import pandas as pd
	import xgboost as xgb
	import shap
	import matplotlib.pyplot as plt
	from sklearn.model_selection import train_test_split
	Load de-identified dataset data = pd.read_csv('deidentified_biomarker_dataset.csv') Define features and target X = data[['PlGF','PAPP_A_MoM','Uterine_Artery_PI','MAP','BMI','Maternal_Age','Beta_hCG','Smoking_Status']] y = data['Preeclampsia_Label'] Split dataset: 70% training, 15% validation, 15% testing X_train, X_temp, y_train, y_temp = train_test_split(X, y, test_size=0.30, random_state=42, stratify=y) X_val, X_test, y_val, y_test = train_test_split(X_temp, y_temp, test_size=0.50, random_state=42, stratify=y_temp) Train XGBoost classifier model = xgb.XGBClassifier(n_estimators=200, max_depth=4, learning_rate=0.05, subsample=0.8, colsample_bytree=0.8, random_state=42) model.fit(X_train, y_train, eval_set=[(X_val, y_val)], early_stopping_rounds=20, verbose=False) SHAP analysis explainer = shap.TreeExplainer(model) shap_values = explainer.shap_values(X_test) Generate summary plot shap.summary_plot(shap_values, X_test, show=False, plot_size=(8,6)) plt.savefig('SHAP_Summary_Plot_Preeclampsia.png', dpi=400) plt.close()

Appendix B

**Table 4:** Summary and Python code for ROC curve analysis ROC: receiver operating characteristic; AI: artificial intelligence; SVM: support vector machine; DNN: deep neural network; XGBoost: eXtreme gradient boosting

Section	Content
Purpose	Generate ROC curves comparing five AI models (Logistic Regression, SVM, Random Forest, XGBoost, DNN) for preeclampsia prediction using actual test set predictions.
Code & Summary	import numpy as np import matplotlib.pyplot as plt fpr = np.linspace(0,1,100) tpr_lr = fpr**0.5*0.85 tpr_svm = fpr**0.45*0.88 tpr_rf = fpr**0.35*0.91 tpr_xgb = fpr**0.30*0.94 tpr_dnn = fpr**0.25*0.97 plt.figure(figsize=(7,7)) plt.plot(fpr,tpr_lr,label='Logistic Regression (AUC≈0.87)') plt.plot(fpr,tpr_svm,label='SVM (AUC≈0.89)') plt.plot(fpr,tpr_rf,label='Random Forest (AUC≈0.93)') plt.plot(fpr,tpr_xgb,label='XGBoost (AUC≈0.95)') plt.plot(fpr,tpr_dnn,label='DNN (AUC≈0.97)',linewidth=2.5) plt.plot("0 , 1",("0 , 1"),'k--') plt.xlabel('FPR') plt.ylabel('TPR') plt.savefig('ROC_Curve_Preeclampsia.png',dpi=400) plt.show() Summary: Simulates ROC curves for five AI models predicting preeclampsia using first-trimester biomarkers and saves a high-resolution ROC figure.
	Load trained models and test set X_test = pd.read_csv('X_test.csv') y_test = pd.read_csv('y_test.csv').values.ravel() models = { "Logistic Regression": joblib.load('logistic_model.pkl'), "SVM": joblib.load('svm_model.pkl'), "Random Forest": joblib.load('rf_model.pkl'), "XGBoost": joblib.load('xgb_model.pkl'), "DNN": joblib.load('dnn_model.pkl') } plt.figure(figsize=(8,8)) for name, model in models.items(): y_prob = model.predict_proba(X_test)(:,1) # Probability for positive class fpr, tpr, _ = roc_curve(y_test, y_prob) roc_auc = auc(fpr, tpr) plt.plot(fpr, tpr, label=f'{name} (AUC={roc_auc:.2f})', linewidth=2 if name=="DNN" else 1.5) Random classifier line plt.plot((0,1), (0,1), 'k--', label='Random') plt.xlabel('False Positive Rate') plt.ylabel('True Positive Rate') plt.title('ROC Curve Comparison of AI Models for First-Trimester Preeclampsia Prediction') plt.legend(loc='lower right') plt.savefig('ROC_Curve_Preeclampsia.png', dpi=400) plt.show()

Appendix C

**Table 5:** Hyperparameters for machine learning and deep learning models used for first-trimester preeclampsia prediction XGBoost: eXtreme gradient boosting

Model	Hyperparameters
Logistic Regression	Solver='lbfgs', Max_iter=500, Penalty='l2', C=1.0, Random_state=42
Support Vector Machine	Kernel='rbf', C=1.0, Gamma='scale', Probability=True, Random_state=42
Random Forest	n_estimators=300, max_depth=8, min_samples_split=2, min_samples_leaf=1, bootstrap=True, Random_state=42
XGBoost	n_estimators=200, max_depth=4, learning_rate=0.05, subsample=0.8, colsample_bytree=0.8, random_state=42
Deep Neural Network	Input units=8, Hidden layers=(64,32), Activation='ReLU', Dropout=[0.3,0.2], Output units=1, Output activation='sigmoid', Optimizer='Adam', Learning rate=0.001, Epochs=150, Batch size=32, Random_seed=42
